# Influence of Type of Prosthesis on Oral Environment and the Number of Missing Teeth in Elderly Persons

**DOI:** 10.1155/2010/584134

**Published:** 2010-09-22

**Authors:** Junko Tanaka, Masahiro Tanaka

**Affiliations:** Department of Fixed Prosthodontics and Occlusion, Osaka Dental University, 1-5-17, Otemae, Chuo-ku, Osaka 540-0008, Japan

## Abstract

The purpose of this study was to investigate the relationship between the number of missing teeth (MT) and the statuses of oral environmental factors (the stimulated salivary flow rate, buffering capacity, and the counts of mutans streptococci, lactobacilli, and *Candida*) in the elderly. The subjects were 64 elderly subjects with fixed prostheses and 49 who wore removable partial dentures aged over 65 years. We used one-way ANOVA to test for overall differences of the number of MT among 5 oral environmental factors. The significant differences were observed in the lactobacilli counts for different number of MT. The number of MT increased with an increase in the lactobacilli counts with removable denture. In conclusion, for the patients wearing removable dentures, increasing number of MT was associated with an increase in the lactobacilli counts in saliva. For the patients with crowns and fixed partial dentures, the number of MT was not significantly affected by salivary mutans streptococci, lactobacilli, and Candida counts.

## 1. Introduction

Caries activity test using saliva is believed to provide useful information for selecting the type of prosthesis. The oral environmental factors considered in the caries activity test include both host and microbial factors associated with caries. We investigated the differences between the statuses of the oral environment factors in elderly individuals with fixed and those with removable prostheses. The results of our study revealed that the amount of cariogenic bacteria in persons with fixed prostheses is different from that of elderly persons with removable dentures [1]. However, there is little evidence to design the prosthesis on the basis of the results of caries activity test. 

The population of the elderly has been increasing worldwide. There is an increase in the number of risk factors of oral diseases and MT in the elderly. The type of prosthesis was decided by the number of MT in most cases. However, the distal extension missing must place removable denture even if the number of MT is little. Therefore, the oral environment should examine not only the statue of the prosthesis, but also the number of missing teeth. To prevent an increase in the number of MT, maintenance of oral environmental factors is valuable and important. 

The purpose of this study was to investigate the relationship between the statuses of oral environmental factors and the number of MT in elderly persons with different types of prosthesis.

## 2. Material and Method

This study protocol was screened and approved for its ethical acceptability by the Committee on Experimental Research on Humans of the Osaka Dental University. 

The volunteers selected included 113 elderly aged over 65 years divided into 2 groups: The volunteers were divided into the crowns and fixed partial denture group (Dentate group), in whom MT were restored only by using fixed partial dentures, and into the removable denture group (Denture group), in whom MT were restored using removable partial and complete dentures.

The one group comprised 64 dentate elderly with fixed prosthesis alone, with a mean age and the number of remaining teeth of 69.8 ± 4.4 years and 26.7 ± 2.8 (Dentate group). The other group comprised 49 elderly with removable prostheses, with a mean age and the number of remaining teeth of 69.4 ± 6.1 years and 15.3 ± 6.6 (Denture group). 

We examined their oral conditions and assessed the oral environmental factors. We selected the following 5 factors for the evaluation of the oral environment: the stimulated salivary flow rate, buffering capacity, and the counts of mutans streptococci (SM), lactobacilli (LB), and *Candida* (CA).

### 2.1. Oral Examination

The number of MT and the kind of prosthesis (fixed partial or removable denture) were determined by oral examination.

### 2.2. Measurement of Oral Environmental Factors

The saliva test was performed using the Dentocult series (Orion Diagnostica, Finland), and saliva was collected more than 1 hour after breakfast, between 9:30 am and 11:30 am. The stimulated salivary flow rate was measured by spitting out saliva after chewing a 1 g paraffin pellet for 5 minutes. The stimulated salivary flow rate was the volume of whole stimulated saliva collected per minute. The stimulated saliva buffer capacity was measured by Dentocult buff strip. The amount of bacteria in the saliva was counted using simple culture kits, Dentocult SM, LB, and CA. After culture, the SM, LB, and CA counts were determined by a comparison with model charts attached to the kits [2–4]. The findings are shown in Table 1.

### 2.3. Statistical Analysis

We examined the relationship between the number of MT and oral environmental factors in individuals with different types of prosthesis (Dentate and Denture group) using one-way ANOVA. The significance level was set to 0.05.

## 3. Results

**Figure 1:**
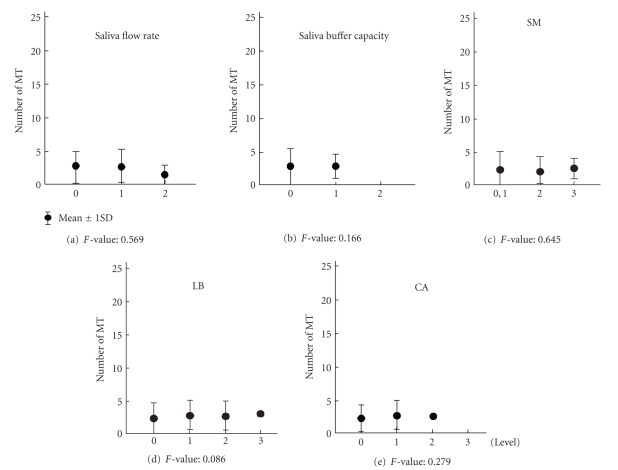
Results of the differences between MT and oral environmental factors in Dentate group.

Figure 1 shows the results obtained for the Dentate group. In the Dentate group, the number of MT for the 5 factors did not vary with increase of the risk level. As a result, there were no differences in the number of MT among 5 factors in the Dentate group.

**Figure 2:**
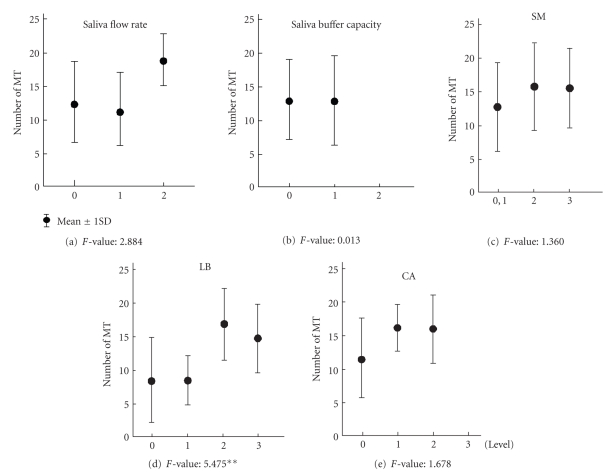
Results of the differences between MT and oral environmental factors in Denture group (**P < .01).

Figure 2 shows the results obtained for the Denture group. In the Denture group, there were no differences in the number of MT among the stimulated salivary flow rate, buffering capacity, and the counts of SM and CA. However, significant differences were observed in the LB counts, and the number of MT was higher with increase of the LB counts.

## 4. Discussion

Dentists should select restorations so as to avoid secondary caries and periodontitis, which will in turn prolong the survival of the remaining teeth. Increase in the number of missing teeth necessitates extensive and large number of restorations. Therefore, it is important to compare the number of MT with the bacterial counts with different types of prostheses.

In general, the volume of stimulated salivary flow is decreased in the elderly. However, the number of MT did not influence the amount of the stimulated salivary flow rate in each group. The results regarding buffer capacity may have been influenced by the saliva volume. The bicarbonate level in saliva is strongly dependent on the amount of saliva secreted. Bicarbonate is one of the main factors determining the buffer capacity of saliva. 

A significant effect on the number of missing teeth was not detected for SM and CA counts in each group. The Dentate group had a lot of low risk level for SM, LB, and CA counts. Therefore, we think that there was no difference. In the Denture group, SM and CA counts have been distributed at each level. However, the number of MT was significantly affected only by LB. LB is associated with dental caries (open carious lesions), poorly executed restorations, and poor oral hygiene [5, 6]. Thus, the high LB count is another risk factor for caries. We have considered that the relationship between the LB count and the number of artificial teeth represented the denture size. Marsh reported an increase in the LB counts due to the presence of dentures [7]. The bacteria may have attached to the rough acrylic resin surface or the inner surface of the dentures in which residual foods easily enter or incompatible regions, such as clasp regions, whereby removal by self-cleansing would be difficult. 

SM increase caries activity in the acid stress [8], whose count does not decrease even after the treatment of caries, and are maintained at a constant level [9]. SM colonize over the smooth surface of the tooth and their elimination by mechanical cleaning is difficult, even when using Professional Mechanical Tooth Cleaning (PMTC) and the chlorhexydine [10, 11].

CA coexist with SM in denture plaques and readily adhere to resin-base dentures [12]. CA counts are high in patients with denture bases and a low saliva volume, resulting in candidiasis [13]; however, denture attachments may not lead to a high risk in healthy persons. 

We think that in routine clinical practice, significant microbial counts should be considered in designing prosthesis. Fixed partial and removal dentures are the 2 main methods for restoration of MT. Removal of dentures is indispensable in cases of a high number of MT, but it is associated with a risk for developing caries. We think that it is preferable to use a fixed partial denture than a removable denture when fixed restoration is possible.

## 5. Conclusion

In conclusion, for the patients wearing removable dentures, increasing number of missing teeth was associated with an increase in the lactobacilli counts in saliva. For the patients with crowns and fixed partial dentures, the number of missing teeth was not significantly affected by salivary mutans streptococci, lactobacilli, and Candida counts.

## Figures and Tables

**Table 1:** Classification of oral environmental factors.

Oral environmental factors	Level			
	0	1	2	3
Stimulated salivary flow rate (mL/min)	≧1.0	0.5 ~ 1.0	<0.5	
Stimulated salivary buffer capacity (pH)	≧6.0	4.5 ~ 5.5	<4.0	
mutans streptococci (CFU/mL)	<1 × 10^5^		1 × 10^5^ ~ 1 × 10^6^	>1 × 10^6^
lactobacilli (CFU/mL)	≦1 × 10^3^	1 × 10^4^	1 × 10^5^	≧1 × 10^6^
Candida (CFU/mL)	≦1 × 10^3^	1 × 10^4^	1 × 10^5^	≧1 × 10^6^

CFU: colony forming units.
